# A Model for Cross-Cultural Reciprocal Interactions through Mass Media

**DOI:** 10.1371/journal.pone.0051035

**Published:** 2012-12-12

**Authors:** Juan Carlos González-Avella, Mario G. Cosenza, Maxi San Miguel

**Affiliations:** 1 Instituto de Física, Universidade Federal do Rio Grande do Sul, Porto Alegre, Brazil; 2 Centro de Física Fundamental, Universidad de los Andes, Mérida, Mérida, Venezuela; 3 IFISC, Instituto de Física Interdisciplinar y Sistemas Complejos (CSIC-UIB), Palma de Mallorca, Spain; University of Zaragoza, Spain

## Abstract

We investigate the problem of cross-cultural interactions through mass media in a model where two populations of social agents, each with its own internal dynamics, get information about each other through reciprocal global interactions. As the agent dynamics, we employ Axelrod's model for social influence. The global interaction fields correspond to the statistical mode of the states of the agents and represent mass media messages on the cultural trend originating in each population. Several phases are found in the collective behavior of either population depending on parameter values: two homogeneous phases, one having the state of the global field acting on that population, and the other consisting of a state different from that reached by the applied global field; and a disordered phase. In addition, the system displays nontrivial effects: (i) the emergence of a largest minority group of appreciable size sharing a state different from that of the applied global field; (ii) the appearance of localized ordered states for some values of parameters when the entire system is observed, consisting of one population in a homogeneous state and the other in a disordered state. This last situation can be considered as a social analogue to a chimera state arising in globally coupled populations of oscillators.

## Introduction

The study of cross-cultural experiences through mass-mediated contact is a topic of much interest in the Social Sciences [1]–[5]. Many of those studies have focused on the effects of cultural product consumption on audience beliefs, emotions, and attitudes toward the group originating these cultural products. For instance, several works have investigated the process by which international audiences develop American values, norms and stereotypes about America through the experience of watching American television series [6]–[8]. Other works have explored the political impact of international television across borders [9]. The expansion of broadcasting and telecommunication industries in recent times has led to an increase in the exchange of mass media products across countries and social groups. As a consequence, people of different groups that may have had little direct contact with each other can, however, have access to their reciprocal mass media messages. For example, the growth of media channels in East Asia has brought changing patterns of cultural consumption: younger generations in China are drawn to Korean pop stars; Korean people have begun to collect Chinese films; Japanese audiences await the broadcast of non-Japanese Asian dramas [5].

In the current research in complex systems, there is also much interest in the investigation of models of social dynamics [10]. Many of these systems have provided scenarios for investigating new forms of interactions and for studying new collective phenomena in non-equilibrium systems [11]–[20]. In this context, the model introduced by Axelrod [21] to investigate the dissemination of culture among interacting agents in a society has attracted much attention from physicists [22]–[33]. In this model, the agent-agent interaction rule is such that no interaction exists for some relative values characterizing the states of the agents that compose the system. This type of interaction is common in social and biological systems where there is often some bound or restriction for the occurrence of interaction between agents, such as a similarity condition for the state variable [34]–[38].

In particular, the effects of local and global mass media on a social group have been studied by using Axelrod's model [26], [27], [39], [40]. Some different formalisms for mass media based on Axelrod's model have also been proposed [41]–[43].

In this paper we investigate the problem of cross-cultural interactions through mass media in a model where two separated social groups, each with its own internal dynamics, get information about each other solely through reciprocal global interactions. We address the question of whether two societies subject to reciprocal mass media interactions become more similar to each other or if they can mantain some diversity. Specifically, our system consists of two populations of social agents whose dynamics is described by Axelrod's model, mutually coupled through global interactions. The global interactions act as fields that can be interpreted as mass media [27], [44]. In our model, the mass media content reaching one population corresponds to the statistical mode or cultural trend originated in the other population, and viceversa.

The existence of non-interacting states in the dynamics, as well as the competition between the time scales of local agent-agent interactions and the responses of the endogenous global fields, lead to nontrivial collective behaviors, such as the emergence of a largest minority group in a population, sharing a state different from that of the applied global field, and the occurrence of localized ordered states. In this last case, one population reaches a homogeneous state while several states coexist on the other. This situation can be considered as a social analogue to a chimera state arising in globally coupled populations of oscillators [45]–[50].

In the next section we present the model for two interacting populations of social agents and characterize the collective behavior on the space of parameters of the system. The nature of the observed localized ordered states is investigated in the following section. The final section contains the conclusions of this work.

## The Model

We consider a system of 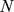 agents consisting of two populations or subsets: 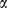 and 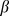, with sizes 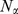 and 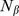, such that 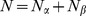. The fraction of agents in subset 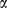 is 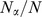 and that in subset 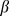 is 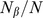.

Each subset consists of a fully connected network, i. e., every agent can interact with any other within a subset. We employ the notation 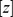 to indicate “or 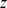
”. The state of agent 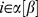 is given by an 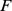-component vector 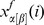, 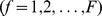, where each component can take any of 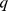 different values 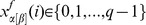.

Let us denote by 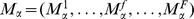 and 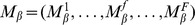 the global fields defined as the statistical modes of the states in the subsets 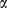 and 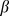, respectively, at a given time. This means that the component 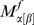 is assigned the most abundant value exhibited by the 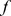
th component of all the state vectors 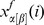 in the subset 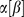. If the maximally abundant value is not unique, one of the possibilities is chosen at random with equal probability. In the context of social dynamics, these global fields can be interpreted as mass media messages about “trends” originated in each population.

**Figure 1:**
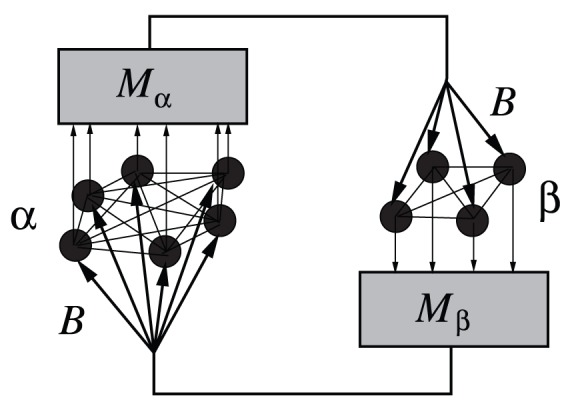
Representation of two populations 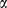 and 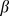 interacting through their reciprocal global fields 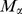 and 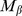, each acting with intensity 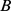.

Each agent in subset 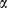 is subject to the influence of the global field 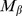, and each agent in subset 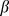 is subject to the influence of the global field 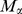. Figure 1 shows the configuration of the two populations subject to the influence of their reciprocal global fields.

Starting from random initial conditions in each subset, at any given time, a randomly selected agent in subset 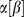 can interact either with the global field 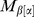 or with any other agent belonging to 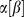. The interaction in each case takes place according to the dynamics of Axelrod's cultural model.

The dynamics of the system is defined by iterating the following steps:

1. Select at random an agent 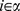 and a agent 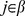.
2. Select the source of interaction: with probability 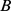, agent 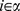 interacts with field 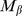 and agent 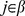 interacts with field 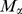, while with probability 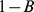, 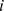 interacts with 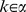 selected at random and 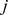 interacts with 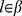 also selected at random.
3. Calculate the overlap (number of shared components) between agent 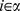 and its source of interaction, given by 
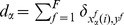
, where 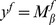 if the source is the field 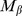, or 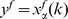 if the source is agent 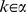. Similarly, calculate the overlap 
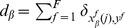
, where 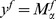 if the source is the field 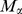, or 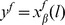 if the source is agent 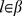. Here we employ the delta Kronecker function, 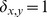, if 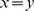; 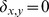, if 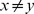.
4. If 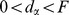, with probability 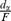 choose 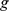 such that 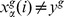 and set 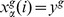; if 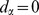 or 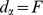, the state 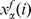 does not change. If 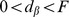, with probability 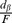 choose 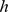 such that 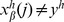 and set 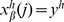; if 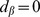 or 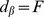, the state 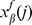 does not change.
5. If the source of interaction is 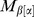, update the fields 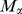 and 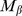.

The strength of each field 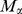 and 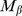 is represented by the parameter 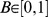 that measures the probability for the agent-field interactions. Step 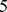 characterizes the time scale for the updating of the global fields in our model. In general, agents in one population do not have instantaneous knowledge of the state of the global field of the other population, but only when they effectively interact with that global field. The non-instantaneous updating of the global fields expresses the delay with which a population acquires knowledge about the other through the only available communication channel between them, as described in many cross-cultural interactions through mass media [5]. In our case, as the value of the parameter 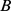 increases, both the intensity of the global fields and the updating rate of their states increase.

Under the mutual coupling, both populations, 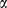 and 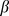 form domains of different sizes in the asymptotic state. A domain is a set of connected agents that share the same state. A homogeneous or ordered phase in a population corresponds to 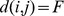, 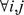. There are 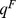 equivalent configurations for this ordered phase. In an inhomogeneous or disordered phase in a population several domains coexist. The sizes of these domains within each population are ranked by the index 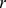: 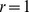 corresponding to the largest domain, 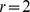 indicates the second largest domain, etc. To characterize the collective behavior of the system, we define the following macroscopic quantities: (i) the average normalized size (divided by 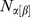) of the domain in 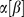 whose size has rank 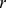, denoted by 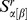; (ii) the probability that the largest domain in 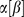 has a state equal to 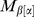, designed by 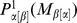.

**Figure 2:**
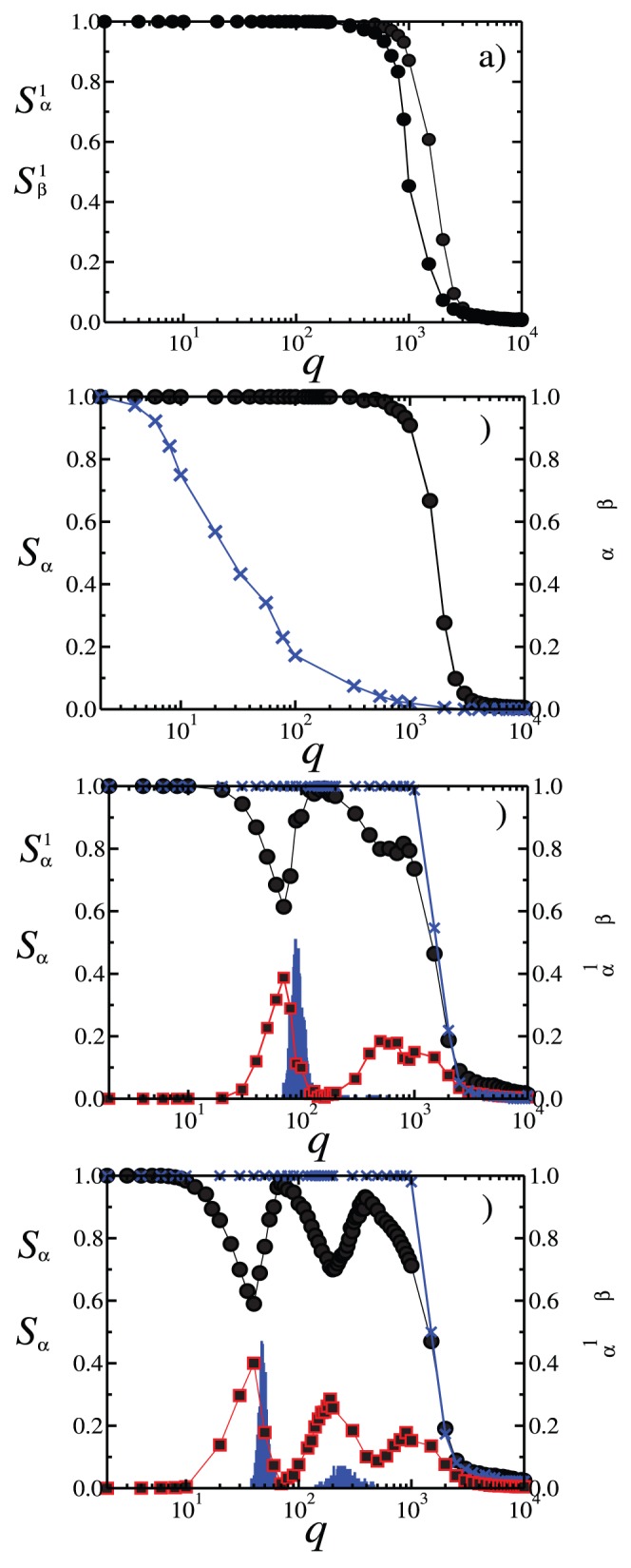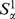, 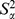, and 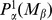 as functions of 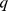, with 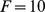, for different values of 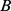. System size is 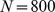 with partition 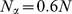. Each data point is the result of averaging over 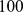 random realizations of initial conditions. (a) 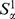 (open circles), 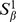 (solid circles); with 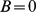. (b) Left vertical axis: 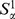 (open circles); right vertical axis: 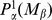 (crosses); fixed 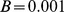. Phases I and II. (c) Left vertical axis: 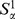 (open circles), 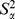 (open squares); right vertical axis: 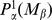 (crosses); fixed 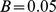. Phases I and IV. (d) Left vertical axis: 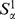 (open circles), 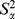 (open squares); right vertical axis: 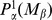 (crosses); fixed 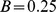. Phase III occurs for values 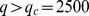, independent of 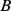. The bars in (c) and (d) indicate the probability 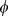 of finding a localized ordered state in the system as a function of 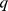 for the given value of the intensity 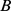.

**Figure 3:**
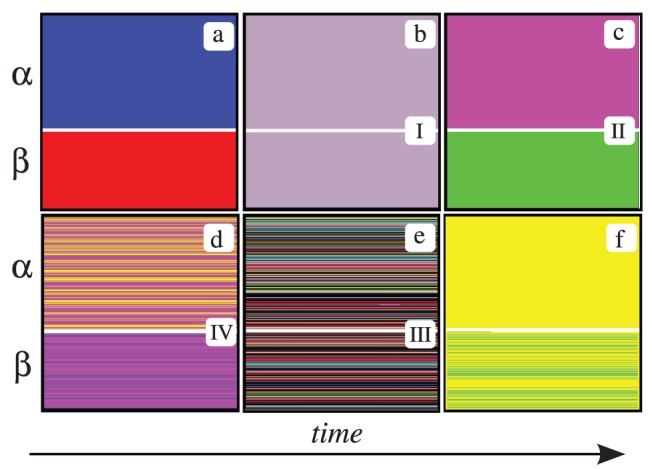
Each panel displays an asymptotic state (vertical axis) of the agents in the interacting populations 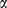 (upper part) and 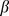 (lower part) vs. time (horizontal axis), corresponding to a different phase in the system. Each value of the state variable of an agent is represented by a different color. Population sizes are 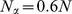, 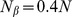, with 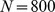. (a) 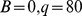 (no coupling). (b) 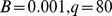 (phase I). (c) 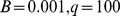 (phase II). (d)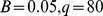 (phase IV). (e) 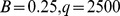 (phase III). (f) 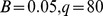 (localized ordered state).

Figure 2 shows various of these quantities as functions of the parameter 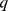, for different values of 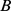. In this paper we fix the parameter value 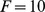. In the absence of global fields (Fig. 2(a)), i.e. 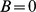, we have two uncoupled and independent subsets; each subset spontaneously reaches an ordered phase, characterized by 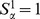 and 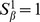, for values 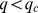, and a disordered phase, corresponding to 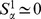 and 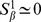, for 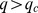, where 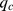 is a critical point that depends on the subset size in each case, 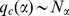 [52]. Figure 3(a) shows the asymptotic pattern in this case.

For 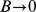 and 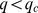, each population reaches an ordered state with 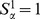, as shown in Fig. 2(b). However, in this situation the spontaneous order emerging in subset 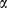 for parameter values 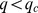 due to the agent-agent interactions competes with the order being imposed by the applied global field 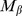. For some realizations of initial conditions, the global field 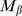 imposes its state on subset 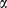 and, correspondingly, the field 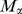 imposes its state on subset 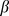. As a consequence, both subsets reach the same state with 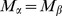. An asymptotic state corresponding to this situation is displayed in Fig. 3(b). We refer to this state as phase I. However, the ordered state in subset 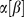 does not always correspond to the state of the global field 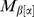 being applied to 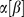. This is revealed by the probability 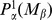 shown in Fig. 2(b) that measures the fraction of realizations that the largest domain in 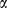 has a state equal to 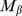. We find 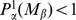 for a range of values 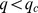. Thus, in this case there is a probability that subsets 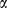 and 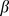 can reach ordered states different from each other, i. e., 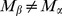. Figure 3(c) illustrates the asymptotic states in this case. We denote this situation as phase II.

Figures 2(c) and 2(d) show both 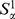 and 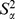 as functions of 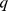 for greater values of 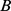. The quantity 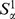 in Fig. 2(c) displays a local minimum at some value of 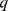 that depends on 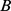. This local minimum of 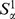 is associated to a local maximum value of 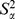, such that 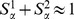 for 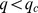. Therefore, two majority domains form in subset 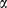 for 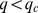. Fig. 2(c) also shows that the probability 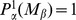, indicating that the state of the largest group in 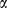 is always equal to that imposed by the field 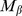. But the second largest group that occupies almost the rest of subset 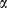 reaches a state different from 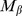. Thus, the value of 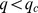 for which 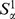 has a local minimum is related to the emergence of a second largest domain ordered against the global field 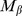. The corresponding asymptotic pattern is shown in Fig. 3(d). We call this configuration phase IV. Figure 2(d) reveals that, for larger values of 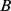, various local minima of 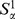 can occur at some values of 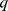. This local minima of 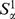 correspond to local maxima of 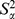 and to the emergence of a second largest domain in 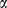 ordered against the field 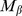. The raise of a largest minority group at some values of 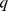 is a manifestation of the tendency towards the spontaneous order related to the agent-agent interactions. For values 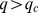, both populations reach disordered states 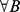, characterized by 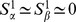. The disordered behavior of the system is denoted by phase III and the corresponding pattern is displayed in Fig. 3(e).

**Figure 4:**
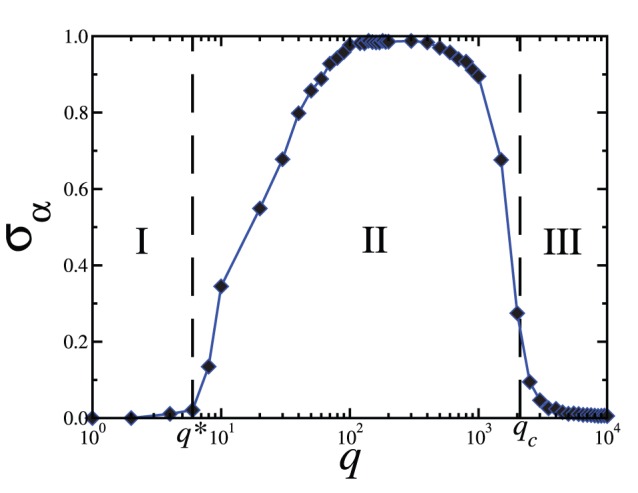
The quantity 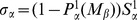 as a function of 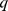 for a fixed value 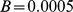, with 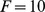. The critical values 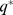 and 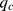, as well as the regions where phases I, II, and III occur, are indicated. System size is 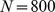 with partition 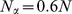. Each data point is averaged over 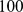 realizations of initial conditions.

To characterize phase II, we plot in Fig. 4 the quantity 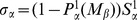 as a function of 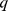, for a fixed value 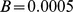. For 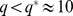, the state of the largest domain in 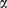 corresponds to the state of the field 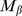, i.e. 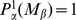 and 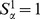, indicating the presence of phase I, and thus 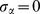. For 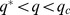, the largest domain in 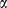 no longer possesses the state of the field 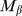 but another state non-interacting with this field, i.e. 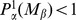 and 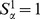, and therefore 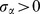, characterizing phase II. For 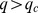, 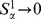 and 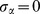.

We note that phase II occurs for small values of 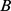, where the time scale for the agent-agent interaction dynamics is smaller than the corresponding time scale for the agent-field dynamics. This means that the state of the global field does not vary much in comparison to the changes taking place in the states of the agents and, therefore, the global field behaves approximately as a fixed external field with little influence on the system. As a consequence the system can spontaneously order in a state different from that of the global field if 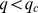 is sufficiently large, giving rise to phase II. For increasing values of 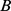, the updating of the global fields and the agent-agent dynamics have comparable time scales and, therefore, the state of the fields corresponds to that of the largest domain in each subset, yielding regions of both phase I and phase IV.

**Figure 5:**
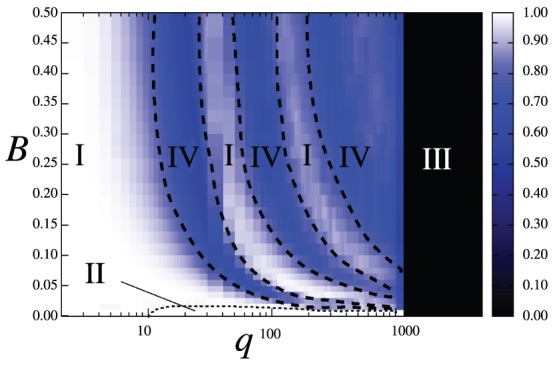
Phase diagram of population 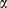 on the space of parameters 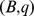, with 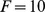. System size is 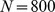 with partition 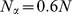. Each data point is averaged over 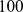 realizations of initial conditions. The color code represents the value of the normalized largest domain size 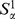, from black (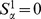) to white (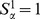). The regions where the different phases occur are labeled and separated by slashed lines: phase I (both populations share same homogeneous state); phase IV (partially ordered, emergence of second group); phase III (disordered), and phase II (each population in a different homogeneous state). Localized ordered states can occur in the transitions from phase IV to phase I.

The collective behavior of either of the two subsets coupled through their reciprocal global fields can be characterized by four phases on the space of parameters 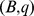, as shown in Fig. 5 for subset 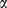: (I) a homogeneous, ordered phase, for which 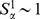 and 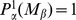; (II) an ordered phase in a state orthogonal to the applied global field, such that 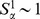 and 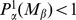; (III) a disordered phase for 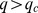, for which 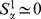; and (IV) a partially ordered phase, where 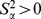 and 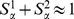, 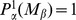, characterized by the emergence of a second largest domain ordered in a state different from field 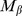.

The phase diagram of Fig. 5 reveals that the interaction through reciprocal, evolving global fields can lead to nontrivial effects in certain cases. For example, for a fixed value 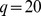, the global field can impose its state to the system (phase I) only for a range of intermediate values of the intensity 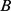.

**Figure 6:**
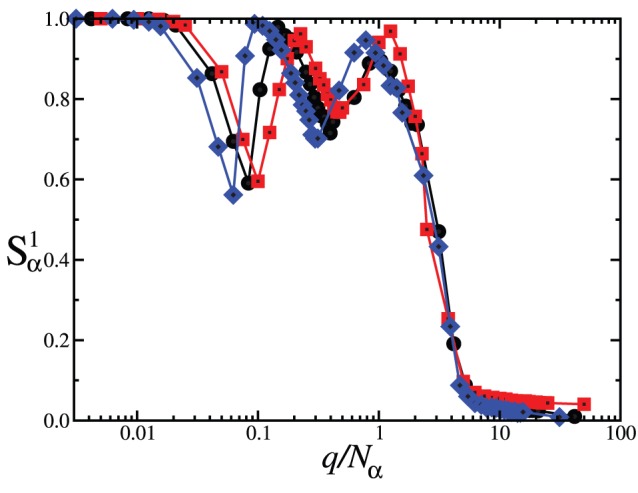
Normalized size of largest domain 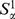 as a function of 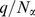 with fixed intensity 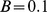, for different population sizes: 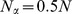 (squares); 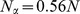 (circles); 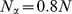 (diamonds). System size is 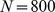 and 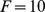.

We have checked the behavior of the system for different population sizes 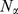 and 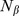. Figure 6 shows the quantity 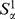 as a function of 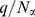 with fixed coupling 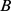, for different values of 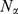. We see that the critical point for the transition to phase III scales as 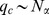, as expected [52], and that the qualitative collective behavior represented in the phase diagram of Fig. 5 is independent of the sizes of the partitions into two populations. Since 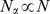, the collective behavior of the system is also independent of the size 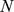, and 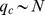, according to Fig. 6.

## Localized Ordered States

In addition to phases I and II that display homogeneous states for both subsets 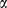 and 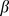, there are configurations where homogeneous states can take place in only one subset, while the other is inhomogeneous, for some values of parameters. We refer to this configuration as *localized ordered states*. These states are characterized by 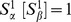 and 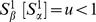. Figure 3(f) displays the asymptotic state of the system in this case. In contrast to the four phases that can be characterized in a subset, the ordered collective states can only be defined by considering both subsets simultaneously, i.e., it requires the observation of the entire system.

**Figure 7:**
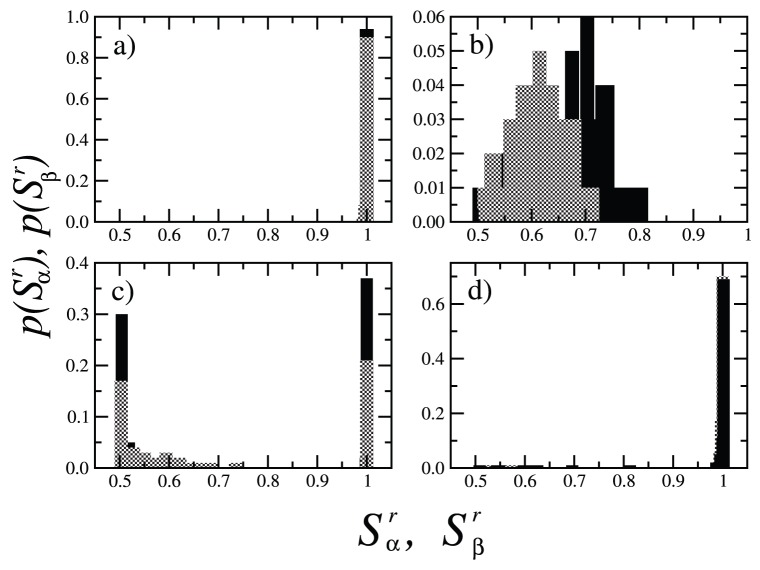
Probability distributions 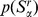 and 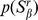, 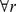, of normalized domain sizes for both populations 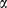 (black bars) and 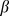 (grey bars), calculated over 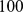 realizations of initial conditions, with fixed intensity 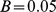, 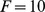, and for different values of the number of options 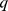. (a) 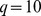 (phase I); (b) 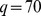 (phase IV); (c) 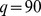 (localized ordered states); (d) 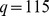 (phase I).

To elucidate the nature of these states, we calculate the probability 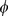 of finding a localized ordered state in the system as a function of 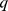 in Figs. 2(c) and 2(d), employing the criterion 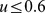. In both figures, there are ranges of the parameter 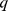 where localized ordered states can occur; the probability 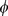 is maximum near the values of 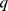 that correspond to local minima of 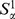 (and local maximum values of 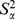). Figure 7 shows the probability distributions 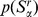 and 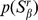, 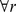, of the normalized domain sizes for both subsets 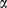 and 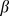, calculated over 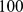 realizations of initial conditions, for different values of 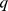, and with fixed 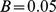 corresponding to Fig. 2(c). Figure 7(a) exhibits the probabilities 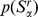 and 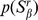 when either subset is in phase I with 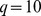, characterized by the presence of one large domain whose size is of the order of the system size 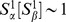, in agreement with Fig. 3(b). Figure 7(b) shows 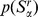 and 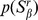 associated to phase IV (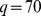), where the size of the largest domain in either subset never reaches the system size due to the appearance of a second group, as displayed in Figs. 2(c) and 3(d). Figure 7(c) shows the probabilities 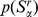 and 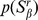 for 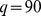. In this case either subset can reach an ordered configuration, 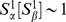, or an inhomogeneous state 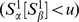. This corresponds to the appearance of localized ordered states in the system. For 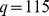, we find again a probability distribution typical of phase I.

The localized ordered states are analogous to chimera states observed in two populations of dynamical oscillators having global or long range interactions, where one population in a coherent state coexist with the other in a incoherent state 45–50. In a chimera state, one part of a spatially extended system presents a coherent or synchronized behavior while another part is desynchronized.

Note that the regions of parameters where localized ordered states can emerge in our system lie between phase IV and phase I states. In fact, the configuration of localized ordered states shares features of both phase I and phase IV; they can be considered as transition configurations between phase IV and phase I states.

## Discussion

We have investigated the collective behavior of a system consisting of two populations of social agents, mutually coupled through global fields, as a model for cross-cultural interactions via mass media. Specifically, we have employed Axelrod's model for social influence as the interaction dynamics.

The global interaction field associated to each population corresponds to the statistical mode of the states of the agents. In the context of social dynamics, this global autonomous field can be interpreted as mass media messages about “trends” or stereotypes originated in one population that are transmitted to the other population. Thus, our system can represent cross cultural interactions between two separated social groups, each with its own internal dynamics, but getting information about each other solely through their mass media messages [5].

We have found several phases on either subset depending on parameter values: two homogeneous phases, one having the state of the global field acting on that subset (phase I), and the other consisting of a state different from that reached by the applied global field (phase II); a partially ordered phase characterized by the emergence of a second largest domain ordered in a state different from the global field (phase IV); and a disordered phase (III).

States similar to phases I, II, and III are also observed for some regions of parameters in a system of social agents subject to an external fixed field [40]. In the present model with non-instantaneous updating of the fields, for small values of 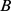, the global evolving field varies very slowly in comparison to the changes in the states of the agents in a subset due to their mutual interactions. In this case, the global evolving field behaves as a fixed external field acting on the population.

However, for larger values of 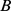, the adaptive nature of the global fields induce two new phenomena in some range of 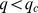 on each population. One is the emergence of a largest minority group of appreciable size having a state different from that of the applied field (phase IV). The other corresponds to the appearance of localized ordered states when the entire system is observed, consisting of one population in a homogeneous state and the other in an disordered state. These configurations occur with a probability that depend on both 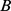 and 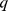 and appear as transitions states from phase IV to phase I. These localized ordered states are analogous to the chimera states that have been found in networks of coupled oscillators having global interactions, where a subset of the system reaches a coherent state while another subset remains incoherent [47], [48]. The recent experimental discovery of such chimera states has fundamental implications as it shows that localized order and structured patterns can emerge from otherwise structureless system [50], [51]. As noted in Ref. [47], analogous symmetry breaking is observed in dolphins and other animals that have evolved to sleep with only half of their brain at a time: neurons exhibit synchronized activity in the sleeping hemisphere and desynchronized activity in the hemisphere that is awake [53].

From a social perspective, our model shows that cross cultural reciprocal interactions through mass media do not always lead to the imposition over one population of the cultural trends being transmitted by the media of another population. A group possessing a cultural state different from that of the mass media message can spontaneously emerge in the first population. Under some circumstances, such group can encompass the entire population (phase II), or it can constitute the largest minority in that population (phase IV).

The behaviors reported here should also be expected in other non-equilibrium systems possessing non-interacting states, such as social and biological systems whose dynamics usually possess a bound condition for interaction [35]. This includes models of motile elements in population dynamics, such as swarms, fish schools, bird flocks and bacteria colonies [34], [54]–[58]. Future extensions of this work involves the consideration of complex network structures within each population and the investigation of communities, where the interaction between populations occurs through a few elements rather than a global field.
