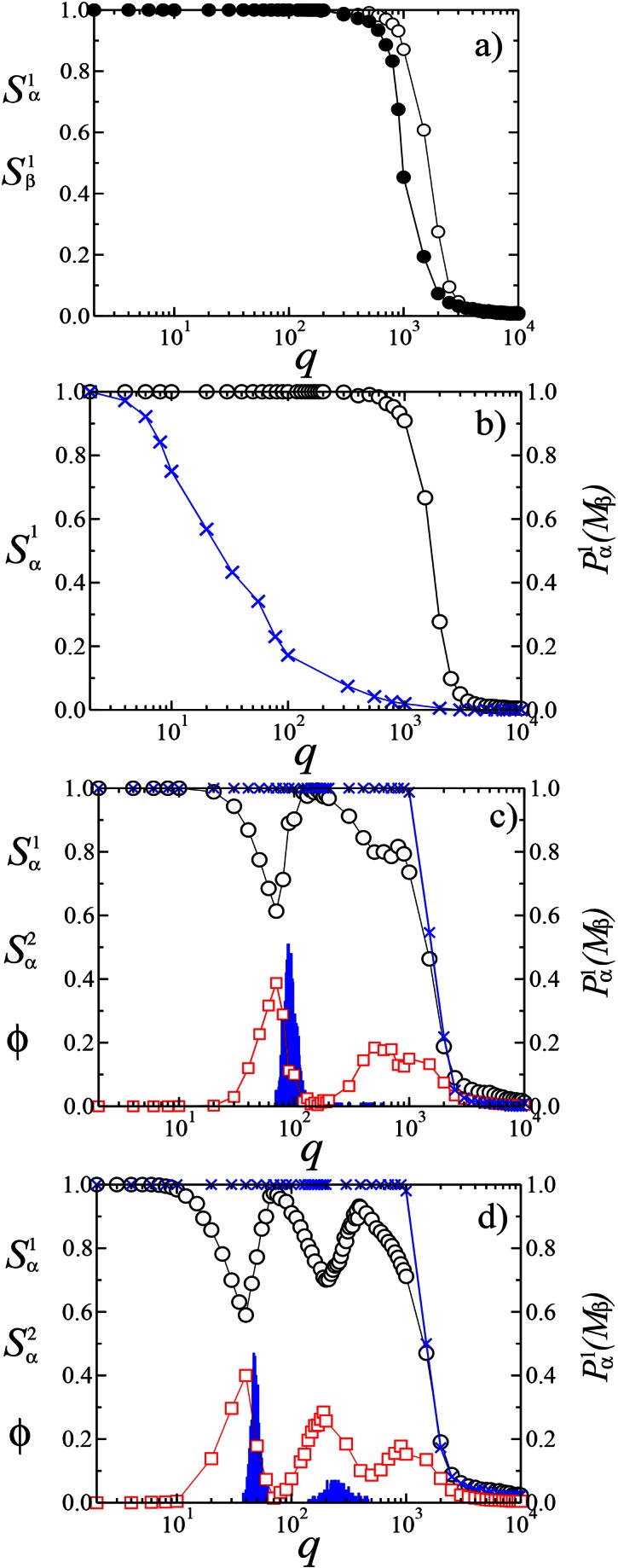# Correction: A Model for Cross-Cultural Reciprocal Interactions through Mass Media

**DOI:** 10.1371/annotation/0870fbde-6809-4cc6-b4af-d65925f806bd

**Published:** 2013-05-22

**Authors:** Juan Carlos González-Avella, Mario G. Cosenza, Maxi San Miguel

There were errors in Figure 2.

The correct version of Figure 2 is available here: 

**Figure:**